# Fruit Quality Attributes of Organically Grown Norwegian Apples Are Affected by Cultivar and Location

**DOI:** 10.3390/plants13010147

**Published:** 2024-01-04

**Authors:** Maja Natić, Dragana Dabić Zagorac, Mihajlo Jakanovski, Anita Smailagić, Slavica Čolić, Mekjell Meland, Milica Fotirić Akšić

**Affiliations:** 1 Faculty of Chemistry, University of Belgrade, Studentski trg 12-16, 11000 Belgrade, Serbia; mnatic@gmail.com; 2 Innovative Centre of the Faculty of Chemistry, University of Belgrade, Studentski trg 12-16, 11000 Belgrade, Serbia; ddabic@chem.bg.ac.rs (D.D.Z.); jakanovski@chem.bg.ac.rs (M.J.); anitasmailagic@yahoo.com (A.S.); 3 Institute for Science Application in Agriculture, Blvd. Despota Stefana 68b, 11000 Belgrade, Serbia; slavicacol@yahoo.com; 4 Norwegian Institute of Bioeconomy Research—NIBIO Ullensvang, Ullensvangvegen 1005, 5781 Lofthus, Norway; 5 Faculty of Agriculture, University of Belgrade, Nemanjina 6, 11000 Belgrade, Serbia; fotiric@agrif.bg.ac.rs

**Keywords:** apples, organic production, geographical origin, biological origin

## Abstract

In this work, 12 apple cultivars grown organically in three regions of Norway (Telemark, Ullensvang, Viken) were analyzed in terms of fruit quality, with the aim of equating different growing regions under specific climatic conditions. Apples were analyzed for concentration levels of minerals, sugars, sugar alcohols, organic acids, total phenolic content (TPC), radical scavenging activity (RSA), and phenolic profiles. Discovery “Rose” from Telemark stored the highest level of minerals (24,094.5 mg/kg dry weight). Glucose, fructose, sucrose, and sorbitol were the major carbohydrates, whereas the predominant organic acids were quinic acid and malic acid. Cultivar Discovery from Ullensvang had the highest TPC (9.22 g/kg) and RSA (229.32 mmol TE/kg). Of the polyphenols quantified, chlorogenic acid and kaempferol-3-*O*-glucoside were the most abounded, accounting for 85.50%. Principal component analysis (PCA) shows that the Ullensvang region is the richest source of most carbohydrates, organic acids (quinic, shikimic, and galacturonic), and most polyphenols, whereas the highest content of minerals and maleic acid characterized Viken. Regardless of location, the Discovery cultivar had, on average, the highest sugar and polyphenol contents. The results obtained suggest that organic apples from Norway are a rich source of beneficial compounds that can have a positive impact on human health. In addition, these results may be useful for consumers in identifying apple cultivars with desirable characteristics and for the fruit industry in tracing back the origin of apples. The findings could also be of great interest for locations with similar climate and soil conditions worldwide.

## 1. Introduction

The apple (*Malus domestica* Borkh.) originated from Central Asia and has made a long journey along the ancient Silk Roads to reach the fertile soils of Europe and North America. It is one of the most important temperate fruit species and is ranked second worldwide (after bananas). Its production is ~93 million tons, with China producing ~46 million tons, which corresponds to 49% of the world’s production [1]. Its large gene pool, successful production in both the northern and southern hemispheres, different appearances, pleasant aroma, and taste, low prices, good transportability, low fruit deterioration, and year-round storage make the apple one of the most popular snack fruits. In addition to fresh consumption, it can also be used for making juices, pastes, jellies, concentrates, marmalades, jams, compotes, teas, wines, dried fruits, ciders, and other products [2]. The nutritional properties, which depend on the cultivar, rootstock, cultural and growing conditions, plant nutrition, storage, and processing, as well as other biotic and biotic factors, especially during fruit ripening, are highly appreciated by consumers and are therefore very popular with consumers [3]. 

Carbohydrates make up to 90% of apple dry matter, its protein content ranges from 1.42% to 4.35%, its total fat ranges from 0.28% to 3.62%, and its ash ranges between 1.32% and 2.08% on a dry matter basis. The most important sugars are glucose and fructose, and it has a low level of sucrose, which makes the apple fruit suitable for consumption by diabetic patients [4]. In addition, apples are a reservoir of other chemicals that belong to primary and secondary metabolism, such as organic acids (malic, maleic, citric, and quinic acid), macro and micro-elements (N, K, P, Na, Ca, Mg, Fe, B, Zn, Cu, and Mn), phenolic compounds (anthocyanins, dihydrochalcones, flavanols, flavones, flavonols, hydroxybenzoic acids, and hydroxycinnamic acids), vitamins (in particular, vitamin C and vitamin E), volatile compounds (esters, alcohols, aldehydes, and ketones), dietary fibers (cellulose, hemicellulose, and pectin), chlorophyll, and carotenoids (lutein, *α* and *β* carotene, neoxanthin, and violaxanthin) [5,6,7,8,9,10,11,12,13]. The seeds, the waste from juice processing, contain up to 27% oils, which are rich in fatty acids, carotenoids, and tocopherols [14]. In addition, apple fruits contain two major (Mal d 1 and Mal d 3) and two minor (Mal d 2 and Mal d 4) allergens that are responsible for most apple allergies in the general population [15]. The seeds contain small amounts of amygdalin (cyanogenic glycoside), a naturally occurring plant toxin, which can reach 3.91 mg/g [16].

Apples are an important part of the diet, and their beneficial characteristics are associated with a reduction in many diseases. The fruits, especially the peels, show antioxidant and antiproliferative activities, inhibit lipid oxidation, and have cholesterol-lowering effects [17]. Apples have been shown to prevent cancer (particularly prostate, liver, colon, and lung cancers), cardiovascular diseases, asthma, Alzheimer’s disease, obesity, and diabetes and improve gastrointestinal health [18]. Apple consumption lowers LDL, inhibits plaque aggregation, and reduces the risk of lung dysfunctions [19,20,21].

Due to the sustainable use of energy, the preservation of ecosystems, the increment of soil fertility, animal welfare, the reduced pollution of water, soil, and air, and the absence of toxic metabolites (heavy metals, synthetic fertilizer, and pesticide residues) in fruits, organic production is in the favor of the consumers [22,23,24]. For this reason, organic fruit management expanded rapidly during the last decade. The apple is the key temperate fruit that accounts for almost 39% of the temperate fruit area, followed by apricots, pears, plums, cherries, and peaches [25]. Global organic apple production in 2020 was ~116,500 ha (2% organic area), with China leading (20,700 ha), followed by France (14,600 ha) and the USA (11,000 ha) [26]. In Norway, the apple is the most important fruit species, with production mainly organized around fjords (western Norway), which are the most northerly fruit-tree-producing areas in the world [8,9,27]. Areas with sufficient heat conditions for apple cultivation increased by 248% from 2011 to 2020 compared to 1971 to 2000. In the period 1971–2000, such areas covered 7750 km^2^, but in the period 2011–2020, it was 26,969 km^2^ [27]. Due to global climate change, it is predicted that the apple will be grown in Norway in the larger inland areas and along the coastline and fjords up to 63.5° N by the end of the century [28]. Organic production in Norway covers more than 45,300 ha, which is 4.6% of the country’s total agricultural land. Organic apple production is performed on 170 ha (11% of organic land) [26]. The demand for organic apples in Norway is high, so the area under organic cultivation is constantly increasing [9].

The area under apples in Norway is increasing and has risen by 15% since 2018 [29]. Apple cultivars that are easy to grow and bear good fruit are early ripening cultivars in the countries south of Norway. The most important commercial cultivars are Discovery, Gravenstein, Summerred, Aroma, Rubinstep, and Elstar [28]. In the past, organic apples from Norway have been studied for their mineral compositions [7], sugars, and polyphenol profiles [9], but those studies have only been conducted in a small area, just around the fjords in Ullensvang. The aim of this study was, therefore, to cover all locations and apple cultivars grown in organic systems throughout Norway. In addition, the cultivars and the three largest growing regions were to be compared in terms of fruit quality in organic apple production in this country.

## 2. Results and Discussion

### 2.1. Elemental Composition

In dried apple samples, 15 elements were detected (Table 1), whereby the contents were predominantly in the range of the values given in the literature for apple fruits [30]. Discovery “Rose” from Telemark (T4) had the highest mineral content (24,094.5 mg/kg dw), which is mainly due to the highest content of K and P. The most abundant element in all samples analyzed was potassium, ranging from 7331.7 mg/kg dw in Discovery from Telemark (T3) up to 21,633.4 mg/kg dw in Discovery “Rose” from Telemark (T4), followed by phosphorus (P), magnesium (Mg) and calcium (Ca). According to the literature data, K is also the most abundant element [31], followed by P [30,32,33]. Similarly, K, Ca, and P were the most abundant elements in Norwegian organic apples grown in western Norway [7]. Previous studies have also shown that the peel of apples has a higher mineral content compared to the mesocarp (especially Ca, Mg, P, and K) [7,34], which means that a significant amount of minerals is lost when an apple is eaten without the peel. The Mg content was higher than the Ca content in all samples analyzed in our study. By contrast, the Ca and Mg content in old Tuscan apple cultivars and the reference cultivar “Golden Delicious” depended on genotype [31]. Moreover, compared to the analysis of apples in Romania [33], the K content in our study was two-fold lower. The Ca content was at the same level, while P, Na, and Mg were much higher in Norwegian organic apples. A possible explanation for this discrepancy could be the production system, the different cultivars, the geographical origin, the climate, and the soil.

The content of micro-elements in the samples examined in this study was more diverse than the content of macro-elements. The most abundant micro-elements were boron (B) and iron (Fe), which is consistent with the results of Šavikin and co-authors [32] for juices, peel, and pulp extracts of indigenous Serbian apple cultivars. The Fe, Mn, Sr, and Zn contents were up to ~6-fold, ~2-fold, ~10-fold, and ~6-fold higher, respectively, than in the study by Todea and co-authors [33], who analyzed conventionally grown international apple cultivars under the agroclimatic conditions of Romania. Our results agree with those of the previous Romanian study with respect to Zn and Cu. Nickel (Ni) and chromium (Cr), on the other hand, were among the least abundant elements in all samples analyzed, with very low levels close to the limit of quantification. Ni was only detected in Red Aroma from Telemark (T1), while Cr was only detected in Red Aroma from Telemark (T1) and Red Elstar from Telemark (T11). Regardless of the locations, Katja had the highest content of Mg, Ca, and Mn, Aroma of B and Al, Holsteiner Cox of Na, and Elstar of Zn. Regardless of the cultivars, the Telemark apple contained the highest levels of K and Cu, the Ullenesvang samples contained Fe and Zn, while the Viken apples stood out as the richest source of all other minerals. This could be because the soil in the eastern part of the country is finer textured and contains more clay, in which more minerals are stored. Of the numerous samples of the Discovery cultivar, U2 had the highest K, Mg, B, and Fe content and should be highlighted as the best. In Rubinstep, T2 had the highest levels of P, K, Mg, Ca, and B, similar to Red Aroma from Telemark—T1.

As far as human health is concerned, minerals are required for every metabolic process (they produce enzymes that act as catalysts, control the action of nerves and muscles, help maintain the body’s water balance, buffer the pH of the cellular and extracellular fluids and are essential for the metabolism of fats, carbohydrates, and proteins), and are therefore necessary for maintaining good health throughout life [35]. Biogenic elements such as P, K, Ca, and Mg are the most important minerals in the human body. Calcium controls the permeability of the cell membrane, plays a role in the contractions of muscle fibers, the transmission of intracellular signals, and the release of hormones, and mediates the nervous system and blood clotting. Phosphorus helps convert food into energy, supports growth and various repair processes in the body, and is involved in the formation of bones, teeth, nucleic acids, and phosphoproteins. Magnesium is involved in calcium metabolism, the synthesis of vitamin D, and the formation of the mineral structure of the bone skeleton. It regulates the activity of more than 300 enzymatic reactions. Potassium controls the pH value, osmotic pressure, and water balance in the body and regulates heart and muscle functions [36,37]. Micro-elements play an important role in the structural fraction of enzymes, in the formation of erythrocyte cells, in the regulation of glucose levels, the activation of antioxidant enzymes, and are involved in the various processes of the immune system [38].

### 2.2. Determination of Sugars and Sugar Alcohols

Sugars are primary metabolic products obtained through the process of photosynthesis. They provide energy and are used as carbon building blocks for biochemical processes. The composition of the sugar is important for the sweetness of the fruit and its acceptance by consumers [39]. The ripening process, the age of the plant, soil characteristics, microclimatic conditions, agro-technical measurements, and the cultivar affect the quantitative variation of sugars in the fruit and can be altered under the influence of biotic and abiotic stress [13,40,41].

Sorbitol plays a fundamental role in plant growth, fruit quality, and adaptation to osmotic stress [42]. Sorbitol is a natural sugar alcohol that increases the water volume in the intestine and contributes to the laxative effects [43]. Sorbitol is slowly absorbed into the body by the gastrointestinal tract and is mainly converted by the liver into fructose, a carbohydrate that is highly tolerated by diabetics [44]. Sorbitol and glucose, which are formed from the products of photosynthesis in leaves, are the translocation sugars that pass through the phloem into the fruit tissue, where they are converted into fructose, malic acid, or starch, depending on the stage of development. Sorbitol is preferentially converted into fructose, while glucose is preferentially incorporated into starch [8,45,46].

In all apple samples analyzed, 8 sugars and 2 sugar alcohols (Table 2) were determined. The predominant sugars in apples were glucose, fructose, and sucrose. After the simple sugars, sorbitol was the most abundant in the apples analyzed. The sorbitol content ranged from 2.32 (Red Aroma from Telemark—T6) to 6.54 g/100 g dw (Rubinstep from Ullensvang—U11). Our results correspond with other studies [13]. The glucose content ranged from 1.92 (Red Aroma from Telemark—T6) to 10.61 g/100 g dw (Discovery from Ullensvang—U4), while the fructose content varied from 10.82 (James Grieve from Telemark—T7) to 31.40 g/100 g dw (Discovery from Ullensvang—U4). With the development of apple fruit, the increase in fructose concentration was accompanied by a decrease in sorbitol concentration, which is consistent with the conversion of sorbitol to fructose [39]. The sucrose content varied between 0.22 (Discovery from Telemark—T3) and 13.56 g/100 g dw (Elstar from Ullensvang—U7).

The percentage of fructose was 40.84–63.36%, glucose 7.92–16.40%, and sucrose 0.96–26.55%, which is consistent with the findings of Queji and co-authors [47] who investigated 26 apple cultivars and obtained 18–31% fructose, 3.4–24% sucrose, and 2.5–12.4% glucose per absolute weight of dry apple pomace. Our results partially correspond with the study of Wu and co-workers [48] for commercial cultivars, who found average values of 53.9% fructose, 33.8% glucose, and 24% sucrose, and with those of Sato and co-authors [49], who found 12.7% glucose, 17.9% fructose, and 7.0% sucrose in different apple cultivars. Most organically grown apple cultivars contain more fructose and less glucose, which is beneficial for diabetic patients as it helps to keep the blood sugar level constant [50]. The Discovery from Ullensvang—U2 had the highest fructose content and almost 3.7-fold lower glucose content, as well as the highest sorbitol content, which provides the sensation of sweetness that is important for diabetes patients. Of the Rubinstep samples, U10 and the Red Aroma (T1) had the best fructose-to-glucose ratio and the highest sorbitol content.

The sum of minor sugars (arabinose, isomaltose, raffinose, maltose, and panose) accounted for between 1.62% (Santana from Telemark—T9) and 9.1% (Rubinstep from Telemark—T2) of the total quantified sugars. Their sum ranged from 0.51 (Discovery “Rose” from Telemark—T4) to 3.75 g/100 g dw (Santana from Ullensvang—U9). Of these, maltose was the most common (average 0.98 g/100 g dw), followed by panose (0.52 g/100 g dw). Apple samples from the Ullensvang area had a higher average sugar content (47.51 g/100 g dw) than samples from Telemark (average value of 28.31 g/100 g dw) (Table 2). In addition, the sum of quantified sugar alcohols in apples from Ullensvang (average 5.27 g/100 g dw) was ~1.5 times higher than in apples from Telemark (3.75 g/100 g dw). The apples from Ullensvang had the highest content of almost all sugars, except for the apples from Viken, which were distinguished by a high content of arabinose and maltose. This can be explained by the longer days, the stronger sunlight, and, thus, the higher photosynthesis rate in the western part of the country. Disregarding the place of production, Discovery apples had the highest content of glucose, fructose, mannitol, arabinose, raffinose, and panose, while Elstar apples were characterized by a high level of sucrose, sorbitol, and maltose.

### 2.3. Determination of Organic Acids

Organic acids are responsible for the formation of fruit acidity and, together with sugars, influence the quality and organoleptic perception of the sweetness and aroma of apples [45]. Organic acids also influence the stability, nutritional value, acceptability, shelf life, and sour-to-astringent flavor of apple fruits. They are also important for processing as they influence the gelling properties of pectin [51].

In the apple samples analyzed, malic acid was by far the most common organic acid, followed by quinic, citric, shikimic, fumaric, and maleic acid (Table 3). According to the literature [52,53], malic acid is the most important acid, with values 10 to 100 times higher than the other acids that contribute to the acidity of the fruit. The amount in apple samples varied from 18.17 (Rubinstep from Ullensvang—U12) to 33.3 g/kg dw (Santana from Telemark—T9). Quinic and citric acid together accounted for 85.15% (Rubinstep from Telemark—T2) to 95.59% (Santana from Telemark—T9) of total acids. The results obtained in this study agree with the results of other authors [8,48,54]. The samples from the Ullensvang area had higher levels of organic acids (quinic, shikimic, galacturonic, fumaric, and maleic acid) than the samples from the Telemark area, which contained higher contents of malic and citric acid. The average value of malic acid in apples from Telemark was 21.60 g/kg dw, in Viken 20.25 g/kg dw, while in Ullensvang, it was 15.87 g/kg, which is lower when compared with the study of Castel and co-authors [55]. Apples from Telemark, Viken, and Ullensvang had 0.75, 0.68, and 0.65 g/kg of citric acid, respectively, which is significantly lower compared to the aforementioned authors. Galacturonic acid was not found in apples from Telemark, while its content in apples from the Ullensvang area was between 0.08 to 0.52 g/kg dw, so this organic acid can be considered to be a potential marker for this location.

Differences between locations were to be expected, as inter-annual climatic fluctuations during the growing season and soil differences play an important role in the acidity profile [53]. Regardless of the location, the Discovery cultivar was recognized by the highest level of quinic acid, Red Aroma by malic acid, Rubinstep by shikimic acid, and Holsteiner Cox by the content of fumaric and citric acid. The results suggest that Norwegian apples are generally more acidic, which is in line with other authors [8,56].

### 2.4. Results of Total Phenolic Content (TPC) and Antioxidant Capacity (RSA)

The TPC of the dried apple samples ranged from 4.10 (Santana, Ullensvang—U9) to 9.23 g/kg (Discovery, Ullensvang—U1) (Table 4), while the antioxidant capacity ranged from 57.57 (Discovery “Rose”, Telemark—T4) to 229.32 mmol TE/kg (Discovery, Ullensvang—U2) (Table 4), which is in accordance with results of Birtic and co-authors [57]. Of the samples analyzed, the Discovery apple cultivar generally had the highest TPC. In addition, cultivars Discovery “Rose” and Katja also had high TPC values compared to other cultivars, while the value for Santana and Holsteiner Cox was low. The TPC value of the Rubinstep cultivar varied greatly (4.92–8.09 g/kg) and depended on factors such as location, weather, and soil properties. On average, apples from Telemark had the highest TPC, followed by apples from Viken. The highest RSA was found in the Discovery cultivar from Ullensvang—U2. The samples of the Discovery cultivar from Ullensvang had a higher RSA value than the samples of the same cultivar from Telemark. On average, the apples from Ullensvang had the highest RSA value, followed by the Viken location.

The correlation between TPC and RSA was to be expected, as the phenolic compounds usually contribute significantly to antioxidant potential. However, no correlation between these parameters was found in the samples tested. In addition to polyphenols, the Folin–Ciocalteu reagent also oxidizes other compounds with redox properties. Furthermore, the RSA is not only selective for phenolic compounds. Possible reasons are mentioned in the literature, the most important of which is the presence of other compounds that influence the antioxidant capacity, such as vitamins and organic acids. The dependence of antioxidant activity on apple varieties has also been commented on in the past [8]. A good statistically significant positive correlation (*p* ≤ 0.0001) was found for radical scavenging activity (RSA) with shikimic acid (*r* = 0.72) and raffinose (*r* = 0.76) for the apple cultivars studied. Despite the low TPC-RSA correlation, these two spectrophotometric assays were able to indicate cultivars with superior fruit quality. It can also be assumed that the phenolic profiles of the studied cultivars differ, which is emphasized in the following section. 

### 2.5. Polyphenol Profile of Investigated Apple Samples

The target analysis of phenolic compounds included the following classes of polyphenols: phenolic acids (5), flavonols (8), flavan-3-ols (1), and coumarins (1). A total of 15 phenolic compounds were identified in dry apple samples, of which only four flavonols (hyperoside, kaempferol-3-*O*-glucoside, quercetin-3-*O*-rhamnoside, and quercetin) were detected in all apple samples (Table 4).

As expected, the most abundant phenolic compound in all apple samples was chlorogenic acid, which accounted for up to 86.04% (Discovery from Telemark—T3) of all quantified polyphenols, except for Rubinstep from Telemark—T8. The highest content of chlorogenic acid was detected in Discovery from Ullensvang—U4 (815.9 mg kg^−1^). This is in accordance with the results for Jonagold apples [58] and Norwegian apples [8]. In addition, Stan and co-authors [59] observed chlorogenic acid as the dominant compound in dried organic Golden Rush and Topaz apples extracted by conventional, microwave- and ultrasound-assisted extraction. Li and co-workers [60] found that the dominant phenolic compound in apple flesh depended on the cultivar. They detected chlorogenic acid as the dominant phenolic compound in Fuji, Qinguan, Jinshuai, and Changmiou cultivars, while epicatechin was dominant in Qingping, Huahong, and Ruiyang apple flesh. Chlorogenic acid was detected as the dominant phenolic acid in Serbian indigenous apple cultivars [32] but not as the most abundant compound. Kschonsek and co-authors [61] detected chlorogenic acid as one of the most abundant phenolic acids in the peel and flesh of old and new apple cultivars from Germany. The possible explanation for all these observations could be different cultivars, geographical origin, climate, soil, and other biotic factors. The second most abundant phenolic compound was kaempferol-3-*O*-glucoside, with the highest content in Discovery from Ullensvang—U4 (609.2 mg kg^−1^). In the study of Fotirić Akšić and co-authors [9], kaempferol 3-*O*-glucoside was one of the most influential compounds that made differences between organic and conventional apples coming from Ullensvang.

The Discovery from Ullensvang—U4 was characterized by an exceptionally high content of gallic acid, chlorogenic acid, ellagic acid, caffeic acid, catechin, aesculetin, rutin, hyperoside, isorahmetin-3-*O*-rutinoside, and kaempferol-3-*O*-glucoside compared to all other samples. The highest content of isorhamnetin was found in Rubinstep from Ullensvang—U11. The apple cultivar Santana from Telemark (T9) showed the highest amount of isorahmetin-3-*O*-glucoside and quercetin-3-*O*-rhamnozide, while Ingrid Marie from Telemark (T10) had the highest content of quercetin. The highest amount of *p*-coumaric acid was detected in Holsteiner Cox from Viken (V1). On the other hand, Rubinstep from Telemark—T8 contained small amounts of phenolic compounds, including two phenolic acids and three flavonols, at a concentration of up to 1.5 mg/kg per compound. In addition, caffeic acid was not detected in Rubinstep from Telemark—T8. Gallic acid was not detected in four samples from Telemark (cultivars Katja—T5, Ingrid Marie—T10, Red Elstar—T11, and Santana—T9). Rutin and isorhametin-3-*O*-glucoside were not detected in Discovery from Telemark (T3), while catechin, *p*-coumaric acid, isorhamnetin-3-*O*-rutinoside, ellagic acid, isorhamnetin, and aesculetin were not detected in six or more samples. 

Regardless of location, the Discovery cultivar had the highest content of gallic acid, catechin, rutin, isorhametin-3-*O*-rutinoside, isorhametin-3-*O*-glucoside, kaempferol-3-*O*-glucoside, and ellagic acid, while Discovery “Rose” stood out due to the highest level of stored chlorogenic acid, caffeic acid, and *p*-coumaric acid. Viken apples had the highest content of *p*-coumaric acid, and Telemark’s apples had the highest content of quercetin and quercetin-3-*O*-rhamnoside. All other phenolic compounds were the highest in apples from Ullensvang. One explanation for the higher content of polyphenols could be that in the western regions, the days are longer, and the light intensity is higher, so secondary metabolism, including the biosynthesis of polyphenols, is more active. In addition, the temperature fluctuations (day/night) in Ullensvang can lead to stress, so polyphenols play a crucial role in protecting plants from unfavorable temperature conditions.

Some of the least abundant compounds were aesculetin (nd—11.8 mg kg^−1^), *p*-coumaric acid (nd—13.2 mg kg^−1^), and isorahmetin-3-*O*-glucoside (nd—12.2 mg kg^−1^). Aesculetin was only present in four samples (Red Aroma from Telemark—T6, James Grieve from Telemark—T7, Discovery from Ullensvang—U4, and Rubinstep from Ullensvang—U10). Fotirić Akšić and co-workers [8] detected aesculetin only in some Norwegian apples from Njøs, but not from the Ullensvang area, while apples from Njøs were not examined in this study. Some of the possible explanations for these discrepancies could be different apple cultivars, different geographical locations, and different soil types that were investigated. Looking at all Discovery, Rubinstep, and Red Aroma apple samples, U4 (Discovery), U6 (Rubinstep), and U5 (Red Aroma) are the best due to the highest content of almost all quantified polyphenols.

### 2.6. Principal Component Analysis

Statistics is an indispensable tool to facilitate the analysis of a large set of chemical data for numerous cultivars from different locations and to discriminate them in terms of fruit quality. PCA has proven to be very useful in investigations of this type to date. In this study, PCA was applied to four separate data sets (mineral, organic acid, sugar, and polyphenol content) to determine whether differences in chemical composition could be explained by differences in geographical and/or biological origin. This was conducted on the basis that differentiation and classification was not possible when PCA was applied to the combined set of chemical data.

PCA of mineral content showed no significant clustering, with the first three components describing only 57.14% of the total variability. The PCA score and loading plots are presented in Figure S1A,B, respectively. The sample V2 exceeded the limits set by the Hotelling’s T2 ellipse with 95% probability, i.e., it was an outlier. However, even after excluding the outlier, the first three components in the PCA model described only 60.10% of the variability, and there was no significant grouping of samples by geographic or biological origin.

PCA of the individual phenols, TPC and RSA, resulted in a two-component model, which explained 64.71% of the total variance (PC1 and PC2 accounted for 52.92% and 11.79%, respectively). The PCA score plot showed no significant clustering of the apple samples (Figure S1C). Discovery, from Ullensvang—U4, differed from the other samples by having the highest contents of gallic acid, chlorogenic acid, caffeic acid, catechin, aesculetin, rutin, hyperoside, isorhamnetin-3-*O*-rutinoside, kaempferol-3-*O*-glucoside, and ellagic acid and could be considered an outlier (Figure S1D). 

**Figure 1:**
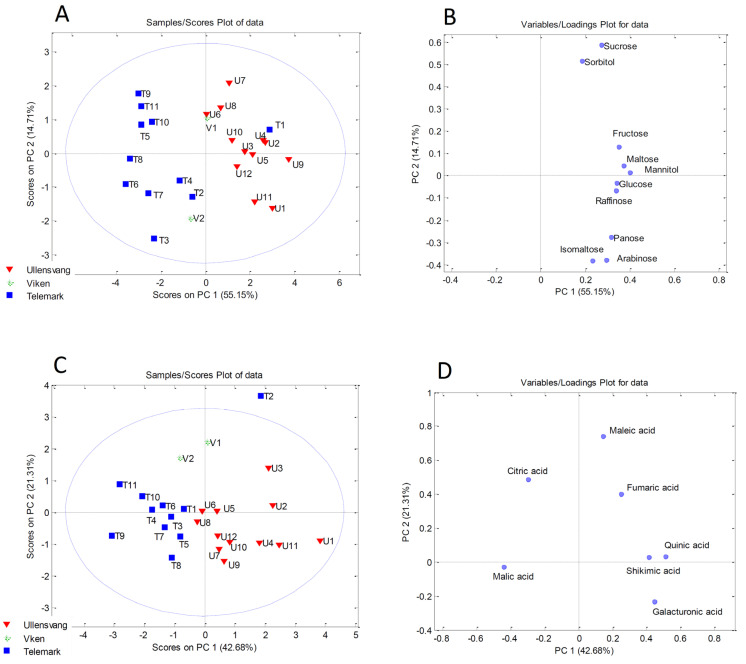
Principal component analysis performed on sugar contents (**A**,**B**) and organic acid contents (**C**,**D**).

PCA applied to the sugar content resulted in a model in which the first three components described 82.21% of the total variability. The first component described 55.15%, the second 14.71%, and the third 12.35% of the variance. The PCA score plot presented in Figure 1A shows the division of the samples into two clusters along the PC1 axis. The samples from Ullensvang were separated from the samples from Telemark due to the higher content of almost all sugars, especially fructose, maltose, mannitol, glucose, and raffinose, as can be seen from the PCA loading plot (Figure 1B). An exception is Red Aroma from Telemark—T1, which was in a cluster with the samples from Ullensvang due to the higher content of individual sugars (Figure 1A).

The best grouping of the samples resulted from the PCA for the organic acid contents. The grouping of the samples into three clusters based on geographical origin can be seen from the PCA score plot (Figure 1C). The only exception was the Rubinstep cultivar from Telemark—T2, which was an outlier. In particular, higher levels of quinic acid, shikimic acid, and galacturonic acid were the most influential variables responsible for the separation of the samples from Ullensvang from the apple samples from the other two locations (Figure 1D). On the other hand, the samples from Viken differed from the other samples due to the higher content of maleic acid.

## 3. Materials and Methods

### 3.1. Climate Conditions

Fruit production in Norway is in the south of the country, where the climate is most favorable, as well as around lakes in the eastern part and in fjord areas in the western part. The fjord areas in western Norway have a maritime climate, with relatively cool summers and mild winters. The weather fronts usually come from the southwest from the North Sea and the Atlantic. There are rarely problems with frost damage to the fruit trees, neither in winter nor during the flowering time. The snow-covered mountains offer protection from large amounts of rain from the west. On the other hand, the climate is the main limiting factor, as the relatively cool summers lead to a relatively short and cool growing season, which limits both the species and the cultivars that can be grown. The climate in Ullensvang (western Norway) in 2021 was slightly warmer and drier than the 30-year climate average (1990–2020). The average annual air temperature that year was 8.2 °C, while the average temperature during the growing season from May to October was 15.1 °C. Total rainfall amounted to 1534 mm, with only 336 mm falling during the growing season. The current climate average (1990–2020) is 7.6 °C and 1705 mm for the year and 12.3 °C and 638 mm for the growing season. 

The climate on the eastern side (Telemark and Viken) is more continental, with warmer summers, colder winters, and less precipitation. Frost can occur in winter and during the flowering season. However, the orchards are mainly located near lakes or by the sea, where the water moderates the more extreme temperatures. In Telemark (Gvarv), the average temperatures in 2021 were 6.8 °C and 15.1 °C for the growing seasons, and the annual rainfall was 663 mm and 369 mm in the growing seasons. The current climate average (1990–2020) is 6.4 °C and 768 mm for the year and 14.0 °C and 399 mm for the growing season.

### 3.2. Plant Material, Soil, and Management

In the fall of 2021, fruit samples of 12 apple cultivars were collected from different commercial organic orchards in the growing areas in western and eastern Norway (Table 5). The organic apple orchards were located at the experimental farm of NIBIO Ullensvang, on the grower side (60.254380, 6.571466; and 60.576515, 6.919554) and in the counties of Telemark (59.374658, 9.221676) and Viken (59.407351, 10.659536), eastern Norway. The locations of these orchards are typical of the regions of Norway’s most important fruit-growing areas. The soil in the Ullensvang area consists mainly of moraines left by the glaciers after the last glaciation 10,000 years ago. It has a high stone content but is favorable for fruit growing as it is rich in minerals and humus but has limited water capacity. The soils are sandy-loamy and very uniform in their morphological and physical characteristics (color and structure). In the orchards in eastern Norway, the proportion of clay soils is higher. Soil composition, organic matter content, CEC (Cation exchange capacity) values, pH, nutrient concentrations, and plant-available nutrients are monitored in these areas [62].

In the Nordic countries, high annual precipitation and a cool climate have led to an accumulation of organic matter in the soil. In Norwegian cultivated mineral soils, a proportion of 5–6% organic matter is normal. In orchard soils, the organic matter is often due to the addition of compost or other organic fertilizers [63]. Organic matter contains a considerable amount of total nitrogen, and mineralization can lead to a significant supply during the growing season [64].

The apple cultivars were grafted onto M9 rootstocks at a spacing of 1.0 × 3.5–4 m. All tested cultivars are tolerant to the main disease, apple scab. The trees were trained as spindle trees and pruned to a maximum height of about 2.5–3 m. The selected trees were homogeneous in terms of flower set, vigor, and health status in all orchards. The organic sites were officially certified by the Norwegian inspection body Debio (Bjørkelangen, Norway) in accordance with the Norwegian regulations on the production and labeling of organic agricultural products.

Organic plant protection was carried out in all orchards in accordance with the official guidelines. The weeds under the trees were removed by frequent mowing and the use of a rotary tiller. The trees were treated several times during the season with sulfur (trade name Thiovit Jet with 80% sulfur as an active ingredient, Syngenta, Frankfurt, Germany), baking powder (sodium bicarbonate), or copper oxide (Trade name Nordox 75WG with 86% copper oxide as an active ingredient) against apple scab. The trees were fertilized with organic chicken manure (pellets), 8% N, 4% P, and 5% K as a percentage of dry matter. Drip irrigation was installed in all fields with a drip line along the tree rows with 0.5 m drip spacing. The trees were watered regularly when a water deficit occurred due to evaporation and precipitation. On average, 2–3 mm of water was applied daily in this relatively cool climate, depending on the evaporation rate. All trees received the same amounts of fertilizer based on soil analysis. Hand thinning was carried out at the end of June to achieve an optimal crop load with good fruit quality (15 cm apart between fruitlets/fruits).

### 3.3. Reagents and Standards

The sugar standards (glucose, fructose, sucrose, arabinose, isomaltose, raffinose, maltose, and panose) were purchased from Supelco/Sigma–Aldrich (St. Louis, MI, USA). Standards for organic acids (quinic acid, shikimic acid, galacturonic acid, fumaric acid, malic acid, maleic acid and citric acid) and polyols (sorbitol and mannitol) were purchased from Supelco/Sigma–Aldrich (St. Louis, MI, USA), while sodium acetate trihydrate and 50% sodium hydroxide solution were from Sigma–Aldrich (St. Louis, MI, USA). All aqueous solutions were prepared with ultrapure water (0.055 µS/cm) obtained using the Thermo Fisher (Waltham, MA, USA) TKA MicroPure water purification system. Polyphenol standards (gallic acid, chlorogenic acid, catechin, caffeic acid, aesculin, rutin, *p*-coumaric acid, hyperoside; isorhamnetin-3-*O*-rutinoside, isorhamnetin-3-*O*-glucoside, kaempferol-3-*O*-glucoside, quercetin-3-*O*-rhamnoside, ellagic acid, quercetin, isorhamnetin) were purchased from Sigma–Aldrich (St. Louis, MI, USA).

### 3.4. Determination of Sugars and Sugar Alcohols by IC

A high-performance anion-exchange liquid chromatography system with pulsed amperometric detection was used to analyze sugars and sugar alcohols. The chromatographic measurement was performed using the Dionex ICS 3000 DP LC system (Dionex, Sunnyvale, CA, USA), which is equipped with a quaternary gradient pump and electrochemical detector consisting of Au as the working electrode and Ag/AgCl as the reference electrode, an autosampler (AS-DV) and Chromeleon software (Chromatography Workstation and Chromeleon Chromatography Management Software—version 6.7). All separations were performed on the Carbo Pac PA100 column (4 × 250 mm (analytical) and 4 × 50 mm (guard); Dionex) thermostatted to 30 °C. The mobile phase flow rate was 0.7 mL/min, and the composition of mobile phase was changed during the analysis in the following order (gradient elution): −20–5 min = 15% 300 mM NaOH; 5–12 min = 15% 300 mM NaOH and 2% 500 mM NaOAc; 12–20 min = 15% 300 mM NaOH and 4% 500 mM NaOAc; 20–30 min = 20% 300 mM NaOH and 20% 500 mM NaOAc; the rest up to 100% is ultrapure water. The total running time of the analyses was 30 min.

### 3.5. Determination of Organic Acids by IC

The organic acid analysis was performed on a Dionex ICS 3000 equipped with a single-channel pump, a conductivity detector and suppressor (DRS 600, Dionex, Dynamically Regenerated Suppressor), an eluent generator (EGC III KOH RFIC), autosampler (AS-DV) and Chromeleon software (Chromatography Workstation and Chromeleon 6.7 Chromatography Management Software). All separations were performed on the IonPac AS15 (4 × 250 mm) analytical column and the IonPac AG15 guard column (4 × 50 mm), which was thermostatted to 30 °C. The flow rate of the mobile phase was 1.0 mL/min, and the composition of the mobile phase was changed during the analysis in the following order (gradient elution): 0–4 min = 10 mM KOH; 4–25 min = from 10 mM to 60 mM KOH (ramp); 25–35 min = from 60 mM to 10 mM (ramp); 35–45 min = 10 mM. The total run time of the analyses was 45 min.

### 3.6. Preparation of Sample Extracts

The extraction of polyphenols from dry apple samples was largely carried out according to the method previously described in the literature [9]. The extraction of polyphenols from ground dry apples (0.5 g) was made with 25 mL methanol/water solution (70/30, *v*/*v*) containing 0.1% HCl using an ultrasonic bath for 30 min. After centrifugation for 10 min at a frequency of 9000 rpm, the supernatant was collected. The extraction procedure was repeated two more times. All supernatants for each sample were combined and evaporated to dryness (IKA RV8 (IKA^®^—Werke GmbH & Co. KG, Breisgau-Hochschwarzwald, Germany). The residue after evaporation was dissolved in 25 mL methanol/water solution (60/40, *v*/*v*) and used for the determination of polyphenol profiles and TPC and RSA analysis.

Sugars, sugar alcohols, and organic acids were extracted according to the procedure described by Fotirić Akšić and co-authors [9]. A total of 0.5 g of each sample was mixed with 50 mL ultrapure water and sonicated for 30 min. The mixture was centrifuged at 9000 rpm for 20 min, and the supernatant was filtered through a 0.22 µm syringe filter. These solutions were used for the analysis of sugar alcohols, minor sugars, and organic acids. For the determination of glucose, fructose, and sucrose, the solutions were diluted 100-fold. 

For elemental analysis, 0.2 g samples were measured and added to PTFE cuvettes with 6 mL of nitric acid (67–70% TraceMetal Grade) and 1 mL of hydrogen peroxide (30–32%). The samples were then digested in a closed microwave system at 170 °C for 10 min and at 200 °C for a further 15 min (Berghof Speedwave Xpert (Limburg, Germany) microwave digestion system). After the cuvettes had cooled down, the samples were quantitatively transferred into PMP volumetric flasks and diluted to 50 mL with ultrapure water Type 1.

### 3.7. Determination of Total Phenolic Content (TPC) and Radical Scavenging Activity (RSA)

TPC and RSA were determined according to the procedures described in our previous work [9]. TPC amounts were expressed as gram gallic acid equivalent (GAE) per kg of dry weight (DW). RSA results were expressed as mmol Trolox equivalent (TE) per kg of DW.

### 3.8. Determination of Polyphenol Profile Using UHPLC-DAD MS/MS

The quantification of the phenolic compounds in the apple samples analyzed was performed using a Dionex Ultimate 3000 UHPLC system equipped with a diode array detector (DAD) and coupled to TSQ Quantum Access Max triple-quadrupole mass spectrometer (ThermoFisher Scientific, Bremen, Germany). The detailed chromatographic separation conditions (column, mobile phase composition, elution conditions, flow rate) have already been described in the literature [9]. The Xcalibur software (version 2.2) was used for instrument control. The polyphenols were identified by direct comparison with commercial standards, and the concentrations of the individual compounds were estimated by calculating the peak areas and expressed as mg/kg.

### 3.9. Statistics

All results obtained were presented as mean values of three replicates. The experimental data were processed using the Tukey test to determine significant differences (*p* ≤ 0.05) between the mean values. This test was performed using the MS Excel statistical program (Microsoft Office 2016 Professional). PCA was performed using the software package PLS_Tool Box for MATLAB (Version 7.12.0), Budapest, Hungary, as described in our previous paper [23]. All data were group-scaled prior to PCA.

## 4. Conclusions

Fruit quality is the main objective of any breeding program. The aim of this study was to select apple cultivars with superior quality and to initiate an organic breeding program in Norway. Twelve apple cultivars from three different locations in Norway, grown under organic management, were analyzed and compared to distinguish them in terms of fruit quality. Rubinstep is the most widely grown apple cultivar in organic farming, followed by Discovery and Red Aroma, while most apple cultivars were grown in Ullensvang, followed by Telemark. 

Based on the results obtained, the authenticity of the locations and cultivars could be determined. The apples from Ullensvang had the highest sugar, polyphenol, and RSA content, while the apples from Viken had the highest acidity, and the apples from Telemark had the highest average mineral content, all due to the day length, sun exposure, temperature fluctuations, and soil characteristics. Galacturonic acid, which was only present in samples from Ullenesvang, could be used as a potential marker for this region. In terms of cultivars, Discovery fruits drew the attention with the highest sugar, polyphenol, and RSA content. The best Discovery apple was sample U2, and the best Red Aroma was sample T1, as minerals and sugars were highest in both samples. For the Rubinstep cultivar, minerals were highest in sample T2, sugars in U10, and polyphenols in U6.

Due to the broad spectrum of compounds detected and the high content of quantitative polyphenolic compounds, as well as the lower content of sucrose and glucose, these different apple cultivars could be used as functional foods, especially for people with diabetes. In addition, the TPC and RSA values showed that the apple cultivars studied can prevent oxidative reactions triggered by free radicals, maintain health in stressful situations, and slow down the aging process. Moreover, all the cultivars studied are of high quality and can be used for the following breeding program to improve the quality characteristics of apples. From a consumer point of view, this study can help consumers select the cultivar with the most preferable properties, while the industry can trace the apples and determine from which region they originate. All data can also be of great interest for locations with similar climate and soil conditions worldwide.

## Figures and Tables

**Table 1:** Elemental composition of investigated dry apple samples (mg kg^−1^ DW).

Sample ID *	P	K	Mg	Ca	Na	B	Al	Mn	Fe	Cu	Zn	Ni	Cr	Sr	Ba
U1	909.0 ^k^	12,188.2 ^r^	490.9 ^r^	267.0 ^l^	97.0 ^f^	6.0 ^s^	4.3 ^f^	3.3 ^n^	15.5 ^e^	3.7 ^k^	4.7 ^f^	<0.5	<0.5	1.2 ^p^	1.9 ^g^
U2	756.5 ^p^	19,182.3 ^b^	667.3 ^g^	236.9 ^n^	22.2 ^p^	29.4 ^de^	<0.5	5.3 ^d^	23.7 ^b^	3.9 ^i^	1.9 ^l^	<0.5	<0.5	3.2 ^h^	1.7 ^h^
U3	794.8 ^o^	13,739.7 ^n^	495.8 ^qr^	110.3 ^t^	20.3 ^q^	18.6 ^j^	<0.5	3.3 ^n^	18.9 ^c^	4.0 ^h^	1.8	<0.5	<0.5	1.1 ^q^	1.0 ^o^
U4	816.8 ^n^	15,434.0 ^f^	526.3 ^p^	140.4 ^s^	14.0 ^r^	17.6 ^k^	1.3 ^m^	4.8 ^f^	13.2 ^gh^	4.4 ^f^	3.6 ^j^	<0.5	<0.5	2.7 ^k^	<0.5
U5	942.5 ^j^	12,748.4 ^q^	549.8 ^n^	199.3 ^q^	91.3 ^g^	8.1 ^p^	0.9 n	3.2 ^o^	10.9 ^m^	2.7 ^p^	1.3 ^n^	<0.5	<0.5	0.8 ^r^	1.4 ^k^
U6	797.5 ^o^	12,905.4 ^p^	478.7 ^s^	278.9 ^k^	88.9 ^h^	4.4 ^t^	2.0 ^l^	3.3 ^n^	10.7 ^m^	5.0 ^d^	11.3 ^d^	<0.5	<0.5	1.3 ^o^	1.6 ^i^
U7	864.4 ^m^	13,166.6 ^o^	497.4 ^q^	235.6 ^no^	89.0 ^h^	3.2 ^u^	6.3 ^d^	3.1 ^p^	12.8 ^i^	3.3 ^l^	17.2 ^a^	<0.5	<0.5	1.3 ^o^	1.5 ^j^
U8	1083.6 ^e^	14,662.5 ^j^	580.6 ^l^	217.8 ^p^	79.0 ^i^	7.6 ^q^	3.3 ^h^	3.4 ^m^	18.7 ^c^	3.7 ^k^	15.0 ^c^	<0.5	<0.5	1.1 ^q^	1.7 ^h^
U9	896.8 ^l^	12,195.3 ^r^	497.9 ^q^	328.2 ^i^	116.5 ^b^	6.3 ^r^	1.3 ^m^	3.1 ^p^	13.3 ^g^	2.6 ^q^	1.3 ^n^	<0.5	<0.5	1.7 ^n^	2.1 ^e^
U10	962.3 ^i^	15,110.0 ^h^	559.8 ^m^	232.6 ^o^	23.8 ^p^	10.9 ^n^	2.3 ^k^	5.1 ^e^	9.6 ^o^	3.8 ^j^	4.1 ^h^	<0.5	<0.5	2.8 ^j^	3.2 ^c^
U11	966.2 ^i^	14,349.8 ^k^	598.4 ^k^	402.5 ^f^	44.4 ^n^	29.4 ^de^	1.1 ^n^	3.9 ^i^	12.0 ^k^	3.9 ^i^	<0.5	<0.5	<0.5	5.2 ^d^	6.2 ^a^
U12	984.1 ^h^	12,748.3 ^q^	468.2 ^t^	346.4 ^h^	32.7 ^o^	28.8 ^ef^	5.7 ^e^	3.5 ^l^	11.3 ^l^	3.7 ^k^	16.2 ^b^	<0.5	<0.5	6.2 ^bc^	1.2 ^m^
V1	1084.7 ^e^	12,407.4	630.5 ^i^	318.8 ^j^	147.2 ^a^	53.3 ^a^	0.7 ^o^	6.0 ^c^	15.2 ^e^	3.9 ^i^	4.8 ^e^	<0.5	<0.5	1.8 ^m^	1.6 ^i^
V2	1059.0 ^f^	14,713.0 ^i^	695.8 ^e^	397.9 ^g^	106.3 ^c^	34.2 ^b^	6.5 ^c^	7.1 ^b^	10.2 ^n^	4.0 ^h^	4.0 ^i^	<0.5	<0.5	2.8 ^j^	2.0 ^f^
T1	985.0 ^h^	14,019.8 ^l^	541.9 ^o^	199.8 ^q^	77.6 ^i^	20.2 ^i^	9.2 ^a^	4.0 ^h^	22.8 ^b^	3.3 ^l^	1.9 ^l^	0.6 ^a^	0.5 ^a^	3.4 ^g^	1.3 ^l^
T2	1373.0 ^c^	17,983.9 ^d^	730.4 ^c^	436.7 ^e^	69.2 ^j^	32.0 ^c^	2.9 ^i^	3.7 ^j^	14.2 ^f^	4.2 ^g^	<0.5	<0.5	<0.5	9.2 ^a^	2.0 ^f^
T3	599.9 ^r^	7331.7 ^t^	272.7 ^w^	143.7 ^r^	103.4 ^d^	10.8 ^n^	<0.5	1.4 ^s^	4.1 ^p^	5.1 ^c^	<0.5	<0.5	<0.5	1.1 ^q^	0.7 ^p^
T4	1424.2 ^b^	21,633.4 ^a^	709.3 ^d^	200.8 ^q^	68.7 ^j^	22.5 ^h^	2.6 ^j^	4.2 ^g^	17.1 ^d^	7.0 ^a^	1.2 ^o^	<0.5	<0.5	2.9 ^i^	0.6 ^q^
T5	1023.5 ^g^	17,702.0 ^e^	786.4 ^a^	581.4 ^b^	107.1 ^c^	17.8 ^k^	4.4 ^f^	4.8 ^f^	34.7 ^a^	5.3 ^b^	4.4 ^g^	<0.5	<0.5	6.3 ^c^	4.2 ^b^
T6	617.5 ^q^	8234.5 ^s^	291.8 ^v^	200.8 ^q^	39.0	13.6 ^m^	<0.5	2.0 ^q^	4.1 ^p^	2.9 ^n^	<0.5	<0.5	<0.5	2.4 ^l^	1.1 ^n^
T7	1000.6 ^g^	13,899.9 ^m^	615.1 ^j^	461.5 ^c^	63.2 ^l^	16.4 ^l^	6.2 ^d^	4.2 ^g^	13.1 ^h^	4.6 ^e^	1.5 ^m^	<0.5	<0.5	4.8 ^e^	2.8 ^d^
T8	520.7 ^t^	6998.3 ^u^	312.9 ^u^	255.4 ^m^	54.3 ^m^	10.4 ^o^	<0.5	1.5 ^r^	1.0 ^q^	2.3 ^r^	<0.5	<0.5	<0.5	0.5 ^s^	<0.5
T9	1452.0 ^a^	19,085.1 ^b^	679.0 ^f^	603.3 ^a^	101.0 ^e^	30.0 ^d^	7.9 ^b^	3.6 ^k^	11.4 ^l^	2.8 ^o^	3.3 ^k^	<0.5	<0.5	8.7 ^b^	2.0 ^f^
T10	1094.4 ^d^	15,217.7 ^g^	653.0 ^h^	441.3 ^d^	65.8 ^k^	28.4 ^f^	3.4 ^g^	13.2 ^a^	11.4 ^l^	3.1 ^m^	1.9 ^l^	<0.5	<0.5	4.4 ^f^	1.5 ^j^
T11	1331.0 ^c^	18,559.0 ^c^	755.2 ^b^	329.4 ^i^	88.6 ^h^	27.0 ^g^	0.9 ^n^	4.2 ^g^	12.6 ^j^	3.1	<0.5	<0.5	0.5	5.0	1.2

* Sample IDs correspond to the geographical and biological origin of apple samples presented in Section 3. Different letters within the same column indicate statistically significant differences at *p* < 0.05 by Tukey’s test.

**Table 2:** Contents of sugars and sugar alcohols in analyzed apple sample (g/100 g DW).

Sample ID *	Glucose	Fructose	Sucrose	Sorbitol	Maltose	Panose	Mannitol	Arabinose	Isomaltose	Raffinose
U1	7.7 ^c^	29.0 ^d^	8.6 ^i^	3.2 ^o^	1.1 ^e^	0.9 ^ab^	0.35 ^a^	0.04 ^b^	0.08 ^d^	0.76 ^bc^
U2	8.4 ^b^	30.8 ^b^	10.5 ^de^	5.0 ^g^	1.3 ^c^	0.7 ^d^	0.17 ^c^	0.03 ^c^	0.06 ^e^	0.80 ^bc^
U3	7.8 ^c^	27.9 ^e^	9.2 ^g^	4.6 ^i^	1.3 ^c^	1.0 ^a^	0.13 ^d^	0.02 ^d^	0.05 ^g^	0.60 ^d^
U4	10.6 ^a^	31.4 ^a^	9.1 ^h^	5.0 ^g^	1.3 ^c^	0.7 ^d^	0.16 ^c^	0.03 ^c^	0.03 ^h^	0.72 ^c^
U5	5.5 ^f^	30.1 ^c^	12.1 ^c^	3.9 ^kl^	0.2 ^k^	0.8 ^c^	0.16 ^c^	0.02 ^d^	0.10 ^bc^	0.80 ^bc^
U6	3.7 ^m^	17.5 ^k^	10.5 ^de^	5.8 ^c^	1.2 ^d^	0.4 ^g^	0.12 ^d^	0.03 ^c^	0.02 ^i^	0.33 ^f^
U7	4.5 ^j^	27.5 ^e^	13.6 ^a^	5.7 ^cd^	1.4 ^b^	0.3 ^h^	0.13 ^d^	0.02 ^d^	0.03 ^h^	0.45 ^e^
U8	4.4 ^k^	20.0 ^i^	11.3 ^d^	5.4 ^e^	1.3 ^c^	0.4 ^fg^	0.17 ^c^	0.02 ^d^	0.03 ^h^	0.52 ^e^
U9	7.0 ^d^	24.0 ^f^	12.4 ^bc^	5.1 ^f^	1.5 ^a^	0.9 ^b^	0.31 ^b^	0.04 ^b^	0.09 ^c^	0.85 ^ab^
U10	4.6 ^i^	19.8 ^i^	8.1 ^k^	6.5 ^a^	1.2 ^d^	0.8 ^c^	0.05 ^g^	0.03 ^c^	0.07 ^d^	0.90 ^a^
U11	5.4 ^g^	21.3 ^h^	7.2 ^l^	4.7 ^i^	1.4 ^b^	0.9 ^b^	0.17 ^c^	0.03 ^c^	0.14 ^a^	0.90 ^a^
U12	4.4 ^k^	18.2 ^j^	8.3 ^j^	5.8 ^c^	1.2 ^d^	1.0 ^a^	0.06 ^g^	0.03 ^c^	0.10 ^bc^	0.88 ^a^
V1	4.9 ^h^	17.6 ^k^	9.9 ^f^	5.4 ^e^	1.2 ^d^	0.5 ^ef^	0.13 ^d^	0.020 ^d^	0.02 ^i^	0.30 ^f^
V2	3.7 ^m^	15.6 ^l^	4.4 ^o^	3.3 ^n^	1.3 ^c^	0.2 ^i^	0.08 ^ef^	0.05 ^a^	0.07 ^d^	0.11 ^g^
T1	6.4 ^e^	30.4 ^b^	12.7 ^b^	6.0 ^b^	1.3 ^c^	0.8 ^c^	0.09 ^e^	0.02 ^d^	0.15 ^a^	0.90 ^a^
T2	3.1 ^n^	11.7 ^o^	4.4 ^o^	4.8 ^h^	1.2 ^d^	0.7 ^d^	0.08 ^f^	0.02 ^d^	0.11 ^b^	0.26 ^f^
T3	3.7 ^m^	14.5 ^m^	0.2 ^r^	2.9 ^p^	0.4 ^i^	1.0 ^a^	0.03 ^h^	0.02 ^d^	0.05 ^f^	0.05 ^hi^
T4	6.4 ^e^	22.5 ^g^	6.4 ^n^	2.6 ^q^	0.9 ^f^	0.1 ^j^	0.05 ^g^	0.03 ^c^	0.03 ^h^	0.03 ^ij^
T5	4.3 ^l^	21.7 ^h^	6.9 ^m^	3.4 ^m^	0.4 ^i^	<LOD	0.02 ^i^	<LOD	<LOD	0.03 ^j^
T6	1.9 ^s^	11.6 ^o^	3.4 ^q^	2.3 ^r^	0.7 ^g^	<LOD	0.05 ^g^	0.01 ^e^	0.02 ^i^	0.03 ^j^
T7	2.9 ^o^	10.8 ^p^	4.2 ^p^	3.8 ^l^	0.6 ^h^	0.2 ^i^	0.01 ^j^	0.02 ^d^	0.09 ^c^	0.08 ^gh^
T8	2.5 ^q^	11.0	4.2 ^p^	4.0 ^k^	0.3 ^j^	0.4 ^g^	0.02 ^i^	<LOD	0.03 ^h^	0.03 ^j^
T9	2.4 ^q^	12.6 ^n^	10.3 ^ef^	5.0 ^g^	0.4 ^i^	0.1 ^j^	0.02 ^i^	<LOD	0.02 ^i^	<LOD
T10	2.8 ^p^	15.6 ^l^	7.6 ^m^	4.1 ^j^	1.0 ^f^	<LOD	0.06 ^g^	<LOD	0.03 ^h^	0.04 ^hi^
T11	2.2 ^r^	11.6 ^o^	7.7 ^l^	5.5 ^d^	0.6 ^h^	0.2 ^i^	0.02 ^i^	<LOD	0.03 ^h^	0.02 ^k^

* Sample ID corresponds to the geographical and biological origin of apple samples presented in Section 3. Different letters within the same column indicate statistically significant differences at *p* < 0.05 by Tukey’s test.

**Table 3:** Organic acid profile determined in the apple samples (g kg^−1^ DW).

Sample ID *	Quinic	Malic	Shikimic	Galacturonic	Fumaric	Maleic	Citric
U1	13.4 ^a^	12.4 ^no^	0.57 ^ef^	0.41 ^c^	0.60 ^c^	0.33 ^i^	-
U2	9.4 ^c^	20.5 ^f^	0.96 ^ab^	0.42 ^c^	0.33 f	0.54 ^d^	0.47 ^p^
U3	7.0 ^f^	12.6 ^q^	0.58 ^f^	0.42 ^c^	0.60 ^c^	0.61 ^c^	0.62 ^j^
U4	8.1 ^d^	13.8 ^o^	0.55 ^f^	0.44 ^bc^	0.25 ^g^	0.35 ^h^	0.38 ^q^
U5	6.5 ^g^	19.0 ^h^	0.43 ^g^	0.20 ^ef^	0.70 ^b^	0.22 ^n^	0.62 ^j^
U6	3.9 ^p^	18.0 ^j^	0.44 ^g^	0.20 ^e^	0.39 ^e^	0.40 ^fg^	0.53 ^n^
U7	5.2 ^k^	17.0 ^lm^	0.44 ^g^	0.46 ^b^	0.30 ^f^	0.19 ^f^	0.62 ^j^
U8	6.3 ^h^	22.7 ^d^	0.65 e	0.19 ^f^	0.24 ^g^	0.28 ^k^	0.75 ^f^
U9	7.7 ^e^	19.7 ^g^	0.61 ^ef^	0.27 ^d^	0.25 ^g^	0.13 ^h^	0.46 ^p^
U10	5.7 ^j^	13.5 ^p^	0.90 ^c^	0.12 ^g^	0.27 ^fg^	0.15 ^g^	0.59 ^l^
U11	7.7 ^e^	12.3 ^r^	1.02 ^a^	0.52 ^a^	0.36 ^e^	0.15 ^g^	0.68 ^h^
U12	5.0 ^l^	11.4 ^s^	0.81 ^d^	-	0.21 ^g^	0.20 ^o^	0.57 ^m^
V1	4.4 ^n^	16.8 ^m^	0.45 ^g^	0.08 ^h^	0.62 ^c^	0.67 ^b^	0.76 ^f^
V2	3.4 ^r^	23.7 ^c^	0.25 ^l^	-	0.93 ^a^	0.48 ^e^	0.59 ^kl^
T1	4.5 ^m^	18.9 ^h^	0.64 ^e^	-	0.19 ^g^	0.36 ^h^	0.72 ^fg^
T2	10.6 ^b^	9.5 ^t^	0.95 ^b^	-	0.50 ^d^	0.78 ^a^	1.28 ^a^
T3	4.4 ^n^	17.2 ^kl^	0.29 ^jk^	-	0.24 ^g^	0.30 ^j^	0.69 ^g^
T4	3.9 ^p^	22.7 ^d^	0.39 ^h^	-	0.23 ^g^	0.28 ^k^	0.87 ^d^
T5	6.1 ^i^	18.4 ^i^	0.37 ^i^	-	0.15 ^i^	0.23 ^m^	0.60 ^k^
T6	5.3 ^k^	21.8 ^e^	0.20 ^j^	-	0.23 ^gh^	0.41 ^f^	0.66 ^i^
T7	3.5 ^q^	19.0 ^h^	0.33 ^j^	-	0.23 ^g^	0.26 ^l^	0.62 ^j^
T8	3.3 ^s^	14.9 ^n^	0.30 ^jk^	-	0.18 ^h^	0.11 ^i^	0.49 ^o^
T9	1.9 ^u^	30.0 ^a^	0.28 ^k^	-	0.23 ^g^	0.11 ^i^	0.85 ^e^
T10	4.2 ^o^	23.8 ^c^	0.26 ^l^	-	0.22 ^g^	0.35 ^h^	0.95 ^c^
T11	2.0 ^t^	24.5 ^b^	0.33 ^j^	-	0.11 ^j^	0.39 ^g^	1.18 ^b^

* Sample IDs correspond to the geographical and biological origin of apple samples presented in Section 3. Different letters within the same column indicate statistically significant differences at *p* < 0.05 by Tukey’s test.

**Table 4:** Amounts of phenolic compounds (mg kg^−1^ DW), TPC (g GAE kg^−1^ DW), and RSA (mmol TE/kg DW) determined in the apple samples.

Sample ID *	GalA	ChlA	Cat	CafA	Aes	Rut	CouA	Hyp	Iso-Rut	Iso-Glu	Kae-Glu	Que-Glu	EllA	Que	Iso	TPC	RSA
U1	14.0 ^g^	225.4 ^i^	7.3 ^j^	16.6 ^f^	-	0.1 ^s^	11.0 ^c^	9.3 ^j^	-	-	2.8 ^v^	5.0 ^i^	27.1 ^g^	35.2 ^e^	-	9.2 ^a^	227.4 ^a^
U2	13.4 ^h^	448.4 ^c^	21.7 ^b^	12.5 ^i^	-	0.1 ^s^	-	11.5 ^h^	-	0.1 ^n^	5.5 ^r^	12.1 ^d^	24.0 ^h^	35.3 ^e^	-	8.1 ^d^	229.3 ^a^
U3	25.2 ^d^	263.4 ^f^	9.7 ^g^	14.3 ^h^	-	19.5 ^g^	-	8.1 ^k^	1.0 ^f^	0.9 ^h^	65.7 ^f^	1.9 ^n^	27.3 ^g^	8.7 ^s^	-	7.5 ^e^	116.2 ^k^
U4	163.4 ^a^	815.9 ^a^	61.9 ^a^	40.5 ^a^	11.8 ^a^	323.9 ^a^	-	68.9 ^a^	23.2 ^a^	11.6 ^b^	609.2 ^a^	15.9 ^b^	309.5 ^a^	36.6 ^d^	-	7.2 ^f^	164.6 ^d^
U5	34.3 ^c^	239.0 ^h^	12.5 ^e^	10.4 ^k^	-	62.7 ^c^	-	21.9 ^e^	4.1 ^c^	3.5 ^d^	174.9 ^c^	5.4 ^h^	65.3 ^c^	25.5 ^j^	-	5.8 ^o^	138.1 ^j^
U6	52.7 ^b^	238.9 ^h^	16.6 ^c^	15.8 ^g^	7.8 ^b^	85.0 ^b^	5.0 ^j^	32.0 ^c^	6.4 ^b^	4.7 ^c^	254.7 ^b^	8.1 ^f^	93.3 ^b^	24.5 ^k^	-	4.9 ^s^	148.5 ^g^
U7	33.7 ^c^	87.7 ^p^	13.0 ^d^	8.2 ^m^	-	31.8 ^d^	-	24.6 ^d^	2.4 ^d^	1.5 ^f^	86.7 ^d^	13.6 ^c^	48.6 ^d^	20.4 ^m^	-	6.1 ^lm^	155.9 ^e^
U8	10.0 ^l^	81.2 ^r^	-	7.4 ^n^	-	1.4 ^r^	-	7.6 ^l^	-	-	3.5 ^u^	5.1 ^i^	17.4 ^k^	30.5 ^i^	3.1 ^g^	5.4 ^p^	146.3 ^h^
U9	15.3 ^f^	27.4 ^t^	7.2 ^j^	5.4 ^r^	-	1.4 ^r^	-	4.5 ^q^	0.6 ^j^	1.2 ^g^	9.8 ^o^	2.1 ^m^	9.9 ^n^	16.7 ^n^	16.2 ^c^	4.1 ^t^	144.7 ^i^
U10	11.5 ^j^	169.5 ^k^	-	30.2 ^b^	-	26.6 ^f^	-	15.3 ^g^	1.7 ^e^	1.5 ^f^	76.0 ^e^	6.6 ^g^	27.2 ^g^	33.2 ^f^	67.0 ^a^	6.2 ^l^	185.7 ^c^
U11	10.9 ^k^	38.8 ^s^	7.8 ^i^	5.9 ^q^	-	8.3 ^n^	9.3 ^d^	5.5 ^n^	0.7 ^i^	0.7 ^i^	38.9 ^h^	0.9 ^p^	16.3 ^l^	3.4 ^f^	2.4 ^i^	6.3 ^k^	143.7 ^i^
U12	11.3 ^j^	179.3 ^j^	6.7 ^k^	5.5 ^r^	-	3.4 ^q^	-	10.4 ^i^	0.4 ^k^	0.2 ^m^	14.2 ^k^	8.0 ^f^	10.2 ^n^	8.6 ^s^	2.4 ^i^	6.8 ^h^	153.4 ^f^
V1	8.0 ^m^	91.4 ^n^	7.6 ^i^	6.2 ^p^	-	-	13.2 ^a^	6.7 ^m^	-	-	4.5 ^t^	4.4 ^j^	15.8 ^m^	32.1 ^g^	3.7 ^e^	5.3 ^q^	188.4 ^b^
V2	0.8 ^r^	143.8 ^l^	-	10.8 ^jk^	-	15.7 ^h^	6.2 ^g^	10.1 ^i^	0.9 ^g^	1.7 ^e^	22.0 ^j^	3.8 ^k^	-	31.4 ^h^	10.7 ^d^	6.6 ^i^	68.7 ^q^
T1	12.1 ^i^	112.9 ^m^	-	9.8 ^l^	-	6.4 ^p^	-	5.1 ^o^	0.7 ^i^	0.5 ^k^	33.6 ^i^	1.1 ^o^	18.3 ^j^	10.2 ^r^	3.5 ^f^	5.2 ^r^	138.3 ^j^
T2	19.0 ^e^	91.9 ^n^	11.6 ^f^	6.0 ^q^	-	12.2 ^j^	-	20.2 ^f^	0.7 ^i^	0.7 ^i^	43.8 ^g^	10.2 ^e^	23.0 ^i^	14.6 ^p^	3.5 ^f^	6.4 ^j^	157.9 ^e^
T3	1.0 ^p^	433.9 ^d^	-	21.2 ^d^	-	-	10.8 ^c^	7.6 ^l^	-	-	2.3 ^w^	3.8 ^k^	-	23.5 ^l^	0.2 ^n^	9.2 ^a^	65.2 ^r^
T4	3.7 ^n^	451.2 ^b^	-	25.1 ^c^	-	13.5 ^i^	12.2 ^b^	3.4 ^r^	0.6 ^j^	0.7 ^i^	12.6 ^l^	1.9 ^n^	-	15.2 ^o^	1.5 ^j^	9.1 ^b^	57.6 ^t^
T5	-	244.5 ^g^	-	19.3 ^e^	-	10.0 ^l^	6.9 ^f^	7.5 ^l^	0.2 ^l^	0.5 ^k^	10.7 ^n^	3.9 ^k^	-	14.0 ^q^	0.4 ^m^	8.2 ^c^	78.1 ^n^
T6	0.6 ^s^	178.6 ^j^	-	6.6 ^o^	5.0 ^c^	9.1 ^m^	7.8 ^e^	4.7 ^p^	0.8 ^h^	0.7 ^i^	10.5 ^n^	3.3 ^l^	-	16.6 ^n^	2.9 ^h^	7.4 ^ef^	60.1 ^s^
T7	1.4 ^o^	83.2 ^q^	6.1 ^l^	6.8 ^o^	5.0 ^c^	9.4 ^m^	1.7 ^k^	21.6 ^e^	0.2 ^l^	0.6 ^j^	11.9 ^m^	13.5 ^c^	-	3.0 ^t^	0.1 ^o^	6.0 ^n^	82.6 ^m^
T8	0.8 ^q^	-	-	-	-	-	-	0.1 ^s^	-	-	1.5 ^x^	0.8 ^p^	0.1 ^o^	0.2 ^u^	-	8.1 ^d^	71.5 ^o^
T9	-	305.8 ^e^	-	11.1 ^j^	-	27.5 ^e^	7.0 ^f^	36.9 ^b^	4.2 ^c^	12.2 ^a^	5.0 ^s^	29.9 ^a^	16.1 ^lm^	38.5 ^c^	36.3 ^b^	6.1 ^m^	73.1 ^p^
T10	-	89.8 ^o^	8.8 ^h^	5.0 ^s^	-	11.0 ^k^	5.4 ^i^	24.5 ^d^	-	0.4 ^l^	6.6 ^p^	5.5 ^h^	36.8 ^e^	65.3 ^a^	0.8 ^l^	7.5 ^e^	77.2 ^n^
T11	-	82.4 ^q^	8.6 ^h^	3.7 ^t^	-	7.2 ^o^	5.7 ^h^	21.6 ^e^	0.2 ^l^	0.7 ^i^	6.2 ^q^	10.1 ^e^	30.8 ^f^	62.1 ^b^	0.9 ^k^	6.9 ^g^	93.2 ^l^

* Samples ID correspond to geographical and biological origin of apple samples presented in Section 3; Different letters within the same column indicate statistically significant difference at *p* < 0.05 by Tukey’s test; GalA—Gallic acid; ChlA—Chlorogenic acid; Cat—Catechin; CafA—Caffeic acid; Aes—Aesculin; Rut—Rutin; CouA—*p*-Coumaric acid; Hyp—Hyperoside; Iso-Rut—Isorhamnetin-3-*O*-rutinoside; Iso-Glu—Isorhamnetin-3-*O*-glucoside; Kae-Glu—Kaempferol-3-*O*-glucoside; Que-Glu—Quercetin-3-*O*-rhamnoside; EllA—Ellagic acid; Que—Quercetin; Iso—Isorhamnetin; TPC—Total phenolic content; RSA—Antioxidant capacity.

**Table 5:** Geographical and biological origin of apple samples.

Region	Orchard Code	Cultivar
Ullensvang	U1	Discovery
	U2	Discovery
	U3	Discovery
	U4	Discovery
	U5	Red Aroma
	U6	Rubinstep
	U7	Elstar
	U8	Holsteiner Cox
	U9	Santana
	U10	Rubinstep
	U11	Rubinstep
	U12	Rubinstep
Viken	V1	Holsteiner Cox
	V2	Aroma
Telemark	T1	Red Aroma
	T2	Rubinstep
	T3	Discovery
	T4	Discovery “Rose”
	T5	Katja
	T6	Red Aroma
	T7	James Grieve
	T8	Rubinstep
	T9	Santana
	T10	Ingrid Marie
	T11	Red Elstar

## Data Availability

Data are contained within the article and Supplementary Materials.

## Supplementary Materials

The following supporting information can be downloaded at: https://www.mdpi.com/article/10.3390/plants13010147/s1. Figure S1: Principal component analysis performed on elemental composition (A,B) and TPC, RSA, and individual polyphenols (C,D). Abbreviations in Figure S1D correspond to polyphenols presented in Table 4.
