# Supplementary material for: Fruit Quality Attributes of Organically Grown Norwegian Apples Are Affected by Cultivar and Location
Source: Plants (Basel). 2024 Jan 4;13(1):147. doi: 10.3390/plants13010147 (PMC10780603; doi:10.3390/plants13010147)
Supplement: Supplementary file 1 [file plants-13-00147-s001.zip › plants-2778440-supplementary.pdf]

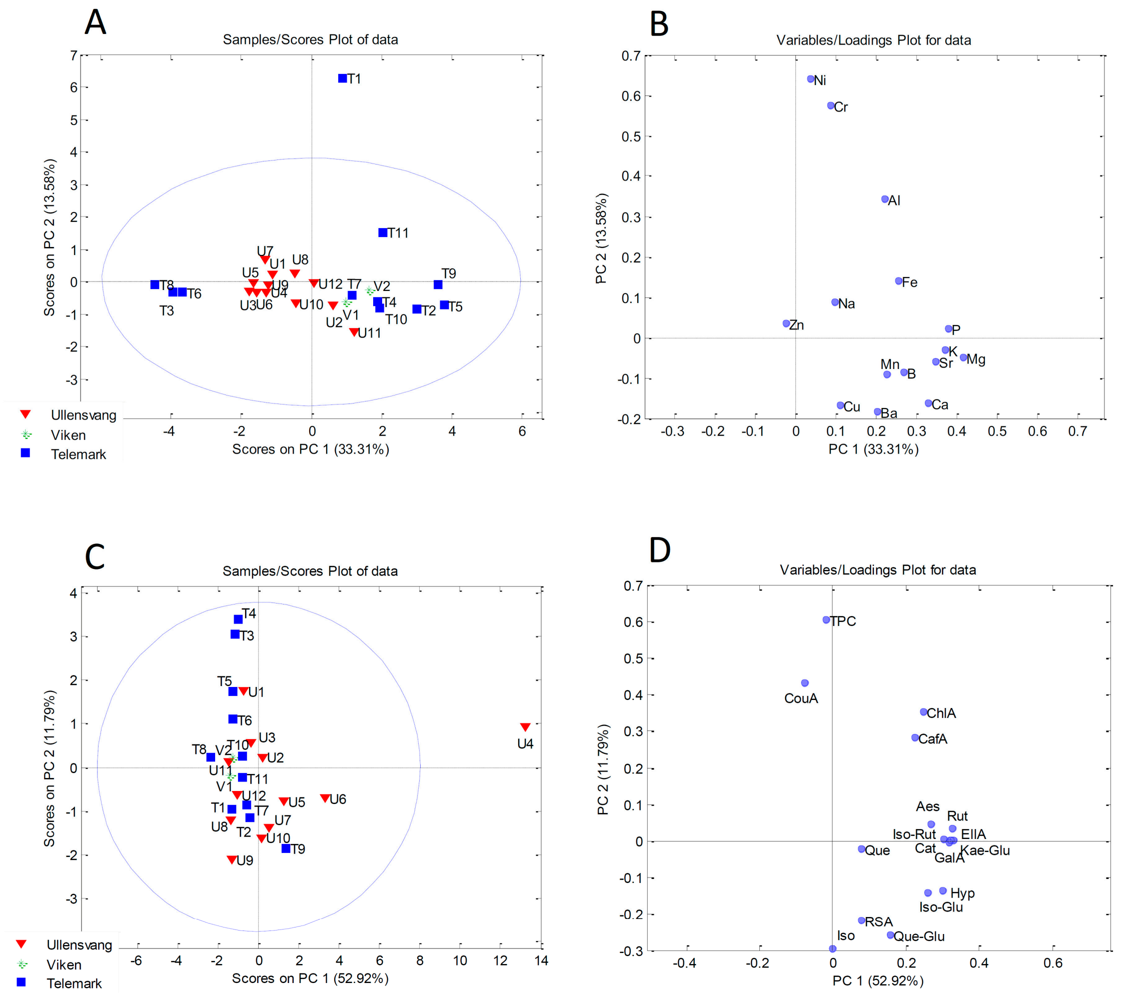

Figure S1. Principal component analysis performed on elemental composition (**A**,**B**) and TPC, RSA and individual polyphenols (**C**,**D**). Abbreviations in Figure S1D correspond polyphenols presented in Table 4.
